# Psoriatic arthritis with hyperuricemia: more peripheral, destructive, and challenging to treat

**DOI:** 10.1007/s10067-022-06061-x

**Published:** 2022-01-20

**Authors:** L. Widawski, T. Fabacher, L. Spielmann, JE. Gottenberg, J. Sibilia, PM. Duret, L. Messer, R. Felten

**Affiliations:** 1 Rheumatology Department, Colmar Civil Hospital, 39 Avenue de la Liberté, 68024 Colmar Cedex, France; 2 Department of Epidemiology and Public Health, Strasbourg University Hospital, 1 Place de l’Hôpital, 67000 Strasbourg, France; 3 Rheumatology Department, Centre National de Référence des Maladies Auto-immunes Systémiques Rares RESO, Strasbourg University Hospital, 1 Avenue Molière, 67098 Strasbourg, France; 4 UMR 7367, MISHA, Allée du Général Rouvillois, Dynamiques Européennes, 67083 Strasbourg Cedex, France

**Keywords:** Crystal arthropathies, Gout, Hyperuricemia, Psoriatic arthritis, Psout

## Abstract

**Objective:**

To study the impact of hyperuricemia on clinical presentation, severity, and associated comorbidities of psoriatic arthritis (PsA).

**Methods:**

Retrospective bicentric case–control study performed in Strasbourg and Colmar, France, from 2009 to 2019. Patients with PsA (according to ICD-10 coding) and at least one available serum urate (SU) measurement were included. Demographic, comorbidities, clinical, and radiographic data were collected. Hyperuricemia was defined as SU level ≥ 360 µmol/L.

**Results:**

We included 242 patients: 73 (30.2%) had hyperuricemia and 15 (6.2%) met 2015 ACR/EULAR criteria for gout. On univariate analysis, as compared with normo-uricemic patients, hyperuricemic patients were more frequently male (72.6% vs 39.1%, *p* = 1.6 × 10^−6^) with higher body mass index (30.9 vs 28.7 kg/m^2^, *p* = 0.015) and more comorbidities (Charlson comorbidity index: 2.6 vs 1.8, *p* = 0.005). PsA started at an older age (47.5 vs 43 years, *p* = 0.016) was more polyarticular (56.2% vs 41.9%, *p* = 0.049) than axial (9.6% vs 22.8%, *p* = 0.019) and more destructive (52.8% vs 37.4%, *p* = 0.032). PsA patients with joint destruction more frequently had hyperuricemia than did others (37.6% vs 25.8%, *p* = 0.047). Multivariable analysis confirmed the association of hyperuricemic PsA with peripheral joint involvement (odds ratio 2.98; 95% confidence interval 1.15–7.75; *p* = 0.025) and less good response to treatment (0.35; 0.15–0.87; *p* = 0.024).

**Conclusion:**

Patients with hyperuricemic PsA show poorer response to PsA treatment and have more peripheral and destructive joint damage than normo-uricemic patients.

**Table:** 

**Key Points** • *Gout and psoriatic arthritis (PsA) can co-exist in the same patient.* • *Monosodium urate crystals might have a deleterious impact on PsA.* • *Hyperuricemic PsA is more polyarticular, less frequently axial, and more destructive than normo-uricemic PsA.* • *PsA with hyperuricemia should lead to more personalized medicine.*

## Introduction

Gout and psoriatic arthritis (PsA) are 2 common diseases that can co-exist in the same patient. These 2 diseases seem strongly linked, but the pathophysiological mechanisms of this link have not yet been defined. Nonetheless, monosodium urate (MSU) crystals play a pathogenic role in psoriasis with a mechanism we could extend to PsA [1]. We hypothesized that a new line of thinking regarding the convergence of gout and PsA in psout, involving MSU crystals, could prompt a potential new approach to treatment (urate-lowering therapy) among patients with active/refractory PsA.

PsA and gout also share comorbidities, both associated with type 2 diabetes, high blood pressure, and increased risk of major adverse cardiovascular events (MACEs) [2, 3]. Metabolic syndrome is associated with both hyperuricemia and PsA [4, 5]. In a large cohort study of 37,315 patients followed for PsA, hyperuricemia was independently associated with cardiovascular disease in PsA patients [6].

Despite examples suggesting that hyperuricemia worsens and maintains skin psoriasis [7, 8], it has not been shown to worsen PsA (leading to more severe, more erosive disease that is more resistant to treatments) or that hyperuricemia is associated with a particular clinical presentation of PsA. The literature contains few observational studies of the effect of hyperuricemia and/or gout on PsA [5, 9, 10], and no study has accurately investigated its clinical presentation and severity. No prospective studies or clinical trials have examined hyperuricemia treatment in PsA. Moreover, we have no recommendations for treating isolated hyperuricemia. Therefore, in the absence of a gout attack history, a patient, even with PsA, should not receive hypo-uricemic therapy [11, 12].

This study aimed to compare PsA patients with normo-uricemia and hyperuricemia. Our main objective was to assess the impact of hyperuricemia on PsA in its clinical presentation, its severity, and associated comorbidities.

## Methods

This was a retrospective case–control, bicentric cohort study. Patients from Strasbourg University Hospital and Colmar Civilian Hospital, France, were included from January 1, 2009, to December 20, 2019.

We included patients with a history of PsA according to their rheumatologists and codes of International Classification of Diseases, 10th edition (ICD-10) and the presence of at least one serum urate (SU) measurement in the medical file. In case of diagnostic doubt with rheumatoid arthritis (e.g., presence of anti-CCP antibodies or rheumatoid factor) during the medical file review, patients were excluded if they met the 2010 ACR/EULAR classification criteria for rheumatoid arthritis.

We excluded patients who expressed their opposition to participate to the study or with a diagnosis other than PsA after the medical file review.

Patients with normo-uricemia and hyperuricemia were compared according to a hyperuricemia threshold ≥ 360 µmol/L, whether they were women or men, based on the median SU level for each patient. To minimize the impact of one isolated hyperuricemia event (e.g., due to an acute renal failure) and to promote the inclusion in the hyperuricemic group with persistent and lasting hyperuricemia, we used the median value for each patient to determine whether they were above this cutoff or not. The median was used to lower sensitivity to extreme values and because of the distribution of these values. This 360-µmol/L cutoff was chosen for several reasons. In vitro, MSU crystals crystallize at 35 °C above the threshold of 360 µmol/L, at which the risk of gout attack is increased in both women and men [13, 14]. The 2015 ACR/EULAR criteria for gout indicate this threshold of 360 µmol/L (60 mg/L), and no point is awarded below this threshold, regardless of sex [15]. Uricemia is an unsteady variable, depending on several factors including renal function [16]. A patient with normo-uricemia may experience hyperuricemia in the event of acute renal failure. To not include the patient in the wrong group (normo-uricemic vs hyperuricemic PsA), we excluded all patients who had hyperuricemia on the basis of a single SU test and who had acute renal failure at the time of the SU test.

Demographic, clinical, biological, and radiological available data were collected. Data collected for PsA included disease duration, age at PsA onset, HLA-B27 status, characteristics of clinical manifestations, and therapeutic history (including conventional synthetic disease-modifying anti-rheumatic drugs [csDMARDs], biologic DMARDs, and targeted synthetic DMARDs). PsA was defined for each patient according to the five Moll and Wright subtypes: oligoarticular, polyarticular, axial, distal, and mutilant [17]. Coexistence of several subtypes was possible. A “good response to ongoing PsA treatment” was defined by the absence of joint flare and elevated CRP level (< 4 mg/L) at the last medical follow-up and if the PsA treatment (csDMARDs, bDMARDs, and/or tsDMARDs) remained unchanged at the last medical appointment. The absence of joint flare was defined by the absence of painful or swelling joint(s) based on clinical examination and/or ultrasonography exploration by the rheumatologist. We were not able to assess PsA with a disease activity index because of the retrospective design of our study. Regarding psoriasis, clinical features of the manifestation and therapeutic history (including psoralene ultraviolet A [PUVA] therapy) were collected.

For each patient, every SU measurement available was collected, as was the number of tests reported, in order to calculate the median SU level for each patient. A history of gout attack or evidence of MSU crystals within a joint was collected. Each patient was tested for 2015 American College of Rheumatology/European League Against Rheumatism (ACR/EULAR) gout criteria. Data on hypo-uricemic and gout attack treatment were collected.

Radiologic characteristics of PSA and gout from standard X-rays, ultrasonography, tomodensitometry, and MRI were collected. PsA was defined as destructive with any erosion related to PsA regardless of the technique (X-ray, ultrasonography, MRI, or CT scan). In addition to comparing hyperuricemic and normo-uricemic patients, we compared patients with destructive and non-destructive PSA.

We collected data on cardiovascular comorbidities, metabolic syndrome (according to the new International Diabetes Federation definition [18]), MACEs, family history of MACEs, history of diabetes, history of moderate to severe chronical renal failure (estimated glomerular filtration rate < 60 mL/min), history of non-alcoholic steatohepatitis, and sleep apnea syndrome. The Charlson comorbidity index was calculated [19]. Toxic exposures such as active smoking and alcohol consumption (> 2 units of alcohol/day) were collected.

In the descriptive part of the analysis, all data collected were summarized as number (percentage) for categorical data and mean (SD), median, and interquartile range (Q1–Q3) for quantitative data. For univariate analysis, the Fisher or chi-square test was used to compare categorical data and Wilcoxon or Student *t* test for quantitative data. The Shapiro test associated with data visualization was used to assess normality. We did not calculate the sample size needed because we included all available patients. We used logistic regression to analyze the primary outcome with estimation of odds ratios (ORs) and 95% confidence intervals (CIs). Model included 7 “hyperuricemia” variables, which were selected according to expert knowledge from the literature, univariate analysis, and backward stepwise selection on the Akaike Information Criterion [20]. To evaluate the impact of hyperuricemia on PsA in its clinical presentation, its severity, and associated comorbidities, we used the same methodology. Goodness of fit of the model was assessed with the Hosmer–Lemeshow goodness-of-fit test. Missing data were assigned by multiple imputation if they did not concern more than 30% of the variable’s data; otherwise, the variables were excluded from the model. *p* < 0.05 was considered statistically significant. All analyses involved using R 4.0.

This study was validated on August 24, 2020, by the Strasbourg University Hospital Ethics Committee (CE-2020–134). Data were anonymized, and then recorded in an Excel table. An anonymous number was established for each patient so that data shared between investigators remained anonymous. Data were stored and analyzed with respect to the rights of all patients.

## Results

We excluded 26 patients after reading the medical file because they did not present PsA. Ten patients had rheumatoid arthritis, 6 exclusives axial spondyloarthritis without psoriasis, 2 SAPHO syndrome, 2 an isolated dactylitis episode without any other articular or extra-articular sign, 3 fibromyalgia without any inflammatory rheumatism, and 1 each isolated osteoarthritis, isolated gout, and isolated skin psoriasis. Within the rheumatology department of the 2 hospitals, 268 patients met our inclusion criteria: 168 in Strasbourg and 74 in Colmar.

The main characteristics of the cohort are in Table 1. The mean age was 58.2 (SD 12.8) years; 50.8% were women; and patients were overweight (mean body mass index [BMI] 29.3 [SD 5.9] kg/m^2^). In total, 34 (30.6%) patients were HLA-B27–positive. The median duration of PsA was 11 [Q1–Q3 6–19] years, and mean age at PsA onset was 44.4 (SD 14) years. Overall, 99 (42.1%) patients had radiographic destructive involvement related to PsA, and 158 (80.3%) showed good response to the last ongoing treatment. As expected, most patients had peripheral articular involvement and 45 (19%) had isolated axial involvement. The mean SU level was 309.2 (SD 88.04) µmol/L. Three (1.2%) patients had a history of MSU crystals in synovial fluid, 15 (6.2%) had a history of gout attack, and 15 (6.2%) met the 2015 ACR/EULAR criteria for gout.

**Table 1:** Characteristics of the cohort (*n* = 242)

Variables	*n* (%)	Median (Q1–Q3)
Age (years)		58 (50–67)
Women	123 (50.8%)	
Men	119 (49.2%)	
BMI (kg/m^2^)		28.78 (25.3–32.9)
HLA-B27–positive	34 (30.6%)	
Age at PsA onset (years)		45.5 (35–53)
Duration of PsA evolution (years)		11 (6–19)
Peripheral PsA	193 (80.4%)	
Isolated peripheral PsA	141 (58.7%)	
Oligoarticular PsA subset	82 (34.2%)	
Polyarticular PsA subset	111 (46.2%)	
Isolated axial PsA subset	45 (18.7%)	
History of dactylitis	65 (26.9%)	
History of enthesitis	35 (14.5%)	
Radiographic destructive PsA	99 (42.1%)	
Positive response to PsA CASPAR criteria	237 (98.3%)	
No treatment	31 (13%)	
NSAIDs alone	7 (2.9%)	
Apremilast alone	9 (3.8%)	
csDMARDs alone	71 (29.5%)	
bDMARDs alone	54 (22.3%)	
csDMARDs + bDMARDs	62 (25.6%)	
Good response to last ongoing treatment	158 (80.2%)	
Number of SU measurements		2 (1–3)
Minimal SU level		276 (224.25–339.8)
Maximal SU level		329.5 (263.75–397.5)
Median SU level		300.25 (246.75–374.4)
Median SU level ≥ 360 µmol/L	73 (30.2%)	
Median SU level ≥ 420 µmol/L	19 (7.9%)	
Evidence of MSU crystals (joint)	3 (1.2%)	
History of gout attack	15 (6.2%)	
Positive response to 2015 ACR/EULAR gout criteria	15 (6.2%)	

*Q1–Q3*, quartile 1 to 3; *BMI*, body mass index; *NSAIDs*, non-steroidal anti-inflammatory drugs; *csDMARDs*, conventional synthetic disease-modifying anti-rheumatic drugs; *bDMARDs*, biologic disease-modifying anti-rheumatic drugs; *PsA*, psoriatic arthritis; *SU*, serum urate; *MSU*, monosodium urate; *CASPAR*, classification of psoriatic arthritis; *ACR/EULAR*, American College of Rheumatology/European League Against Rheumatism

The comparison between patients with hyperuricemia (*n* = 73) and normo-uricemia (*n* = 169) is in Table 2. As compared with normo-uricemic patients, hyperuricemic patients were older (mean 61 vs 57 years, *p* = 0.020) and more frequently men (72.6%, *n* = 53, vs 39.1%, *n* = 66, *p* = 1.6 × 10^−6^). They had higher mean BMI (30.9 vs 28.7 kg/m^2^, *p* = 0.015). The 2 groups did not differ in HLA-B27 positivity. The mean age at PsA onset was significantly higher in the hyperuricemic than normo-uricemic group: 47.5 (SD 13.71) vs 43 (SD 14) years (*p* = 0.016), but the groups did not differ in median duration of PsA. At the end of follow-up, as compared with normo-uricemic patients, hyperuricemic patients had a more peripheral PsA involvement (89%, *n* = 65, vs 76.7%, *n* = 128, *p* = 0.033) and more polyarticular disease (56.2%, *n* = 41, vs 41.9%, *n* = 70, *p* = 0.049). They had significantly less isolated axial involvement of PsA (9.6%, *n* = 7, vs 22.8%, *p* = 0.019). Overall, 38 (52.8%) had radiographic destructive PsA as compared with 63 (38.7%) of normo-uricemic patients (*p* = 0.047). Erosion seen on standard X-rays was more frequent for hyperuricemic than for normo-uricemic patients (43.7%, *n* = 31, vs 28%, *n* = 44, *p* = 0.023). Good response to ongoing PsA treatment was lower for hyperuricemic than normo-uricemic patients (70.7%, *n* = 41, vs 84.2%, *n* = 117, *p* = 0.048), with no difference between the groups in distribution of last ongoing treatments.

**Table 2:** Univariate analysis: principal characteristics of normo-uricemic and hyperuricemic patients with psoriatic arthritis (PsA)

Variables	Normo-uricemic (*n* = 169)		Hyperuricemic (*n* = 73)			*p*
	*n* (%)	Median (Q1–Q3)	*n* (%)	Median (Q1–Q3)		
Age (years)	169	57 (48–66)	73	61 (53–68)		**0.020**
Female	103 (61%)		20 (27.4%)			**1.6 × 10**^**−6**^
Male	66 (39.1%)		53 (72.6%)			
BMI (kg/m^2^)	148	28.3 (24.3–32.2)	64	30 (26.3–34.5)		**0.015**
Psoriatic arthritis						
HLA-B27–positive	24 (32%)		10 (27.8%)			0.83
Age at PsA onset (years)	164	44.5 (32.8–52)	72		49 (39.5–57.3)	**0.016**
Duration of PsA evolution (years)	164	11 (6–19)	72		11 (8–17)	0.85
Peripheral PsA	128 (76.6%)		65 (89%)			**0.033**
Oligoarticular PsA subset	58 (34.7%)		24 (32.9%)			0.88
Polyarticular PsA subset	70 (41.9%)		41 (56.2%)			**0.049**
Isolated axial PsA subset	38 (22.8%)		7 (9.6%)			**0.019**
History of dactylitis	42 (25.1%)		**2**3 (31.5%)			0.34
History of enthesitis	21 (12.5%)		14 (19.2%)			0.23
Radiographic destructive PsA	61 (37.4%)		38 (52.8%)			**0.032**
Last ongoing treatment for PsA						
No treatment	24 (14.5%)		7 (9.6%)			0.40
NSAID alone	4 (2.4%)		3 (4.1%)			0.44
Apremilast alone	4 (2.4%)		5 (6.9%)			0.14
csDMARD alone	48 (28.6%)		23 (31.5%)			0.65
bDMARD alone	40 (23.7%)		14 (19.2%)			0.50
csDMARD + bDMARD	40 (23.7%)		22 (30.1%)			0.34
Good response to last ongoing treatment	117 (84.2%)		41 (70.7%)			**0.048**
Uricemia and gout						
Median SU level	169	274 (227–305)	73		398 (380–420)	**5.5 × 10**^**−5**^
Evidence of MSU crystals	0 (0%)		3 (4.1%)			**0.027**
History of gout attack	2 (1.2%)		13 (17.8%)			**4.6 × 10**^**−6**^
Positive response to 2015 ACR/EULAR gout criteria	1 (0.6%)		14 (19.2%)			**2.3 × 10**^**−7**^

*Q1–Q3*, quartile 1 to 3; *BMI*, body mass index; *NSAIDs*, non-steroidal anti-inflammatory drugs; *csDMARDs*, conventional synthetic disease-modifying anti-rheumatic drugs; *bDMARDs*, biologic disease-modifying anti-rheumatic drugs; *PsA*, psoriatic arthritis; *SU*, serum urate; *MSU*, monosodium urate; *CASPAR*, classification of psoriatic arthritis; *ACR/EULAR*, American College of Rheumatology/European League Against Rheumatism

Bold: *p*<0.05

The study of the main comorbidities is in Table 3. The mean Charlson comorbidity index was significantly higher for hyperuricemic than normo-uricemic patients (2.6 vs 1.8, *p* = 0.005). Significantly more hyperuricemic than normo-uricemic patients had metabolic syndrome (53.4%, *n* = 39, vs 32.5%, *n* = 55, *p* = 0.003), high blood pressure (57.5%, *n* = 42, vs 29.6%, *n* = 50, *p* = 5.3 × 10^−5^), MACEs (16%, *n* = 12 vs 5%, *n* = 8, *p* = 0.004), ischemic stroke (8.2%, *n* = 6, vs 1.8%, *n* = 3, *p* = 0.024), acute coronary syndrome (9.6%, *n* = 7, vs 3%, *n* = 5, *p* = 0.048), moderate or chronic renal failure (15.1%, *n* = 411, vs 3%, *n* = 5, *p* = 0.001), and type 2 diabetes (30.1%, *n* = 22, vs 14.2%, *n* = 24, *p* = 0.007).

**Table 3:** Univariate analysis: differences in main comorbidities between normo-uricemic and hyperuricemic patients with psoriatic arthritis (PsA)

Variables	Normo-uricemic (*n* = 169)		Hyperuricemic (*n* = 73)		*p*
	*n* (%)	Median (Q1–Q3)	*n* (%)	Median (Q1–Q3)	
Charlson comorbidity index	169	2 (1–3)	73	2 (1–3)	**0.0051**
BMI (kg/m^2^)	148	28.3 (24.3–32.2)	64	30 (26.3–34.5)	**0.015**
Metabolic syndrome	55 (32.5%)		39 (53.4%)		**0.0026**
High blood pressure	50 (29.6%)		42 (57.5%)		**5.2 × 10**^**−5**^
MACEs	8 (4.7%)		12 (16.4%)		**0.0043**
Ischemic stroke	3 (1.8%)		6 (8.2%)		**0.024**
Acute coronary syndrome	5 (3%)		7 (9.6%)		**0.048**
Moderate to severe renal failure	5 (3%)		11 (15.1%)		**0.0011**
Type 2 diabetes	24 (14.2%)		22 (30.1%)		**0.0068**

*BMI*, body mass index; *MACEs*, major adverse cardiovascular events

Bold: *p*<0.05

Uricemia was measured more frequently in patients with destructive than non-destructive PsA (mean 4.66 [SD 7.29] vs 3.25 [SD 5.49], *p* = 0.024) and the median SU level was significantly higher (median 321 [Q1–Q3 268–382] vs 288.8 [234–359.1] µmol/L, *p* = 0.0038). Destructive disease was associated with SU level ≥ 300 µmol/L (59%, *n* = 60, vs 45% non-destructive disease, *n* = 60, *p* = 0.037) and ≥ 360 µmol/L (37.6%, *n* = 38 vs 25.8%, *n* = 34, *p* = 0.047).

On multivariable analysis based on the “hyperuricemia” variable (Table 4), variables significantly associated with hyperuricemia in PsA were male sex, high blood pressure, moderate or severe chronic renal failure, PUVA therapy, peripheral PsA involvement, and poor response to PsA ongoing treatment. Probability of peripheral PsA with hyperuricemia was increased threefold (*OR* 2.98, 95% *CI* 1.15–7.75, *p* = 0.025). The probability of a good response to ongoing treatment was reduced for patients with PsA and hyperuricemia (*OR* 0.35, 95% *CI* 0.15–0.87, *p* = 0.024). Radiographical destructive PsA was associated but not significantly with hyperuricemia (*OR* 1.45, 95% *CI* 0.71–2.96, *p* = 0.31).

**Table 4:** Multivariable analysis of variables associated with hyperuricemia in PsA

Variable	*OR*	95% *CI*	*p* value
Male sex	3.78	1.79–8.03	**0.0006**
High blood pressure	2.28	1.17–4.46	**0.016**
Chronic renal failure	7.15	1.83–27.87	**0.0048**
PUVA therapy	2.75	1.06–7.12	**0.037**
Peripheral PsA	2.98	1.15–7.75	**0.025**
Good response to ongoing treatment	0.35	0.15–0.87	**0.024**
Radiographic destructive PsA	1.45	0.71–2.96	0.31

*OR*, odds ratio; *95% CI*, 95% confidence interval; *PsA*, psoriatic arthritis; *PUVA*, psoralene ultraviolet A

Bold: *p*<0.05

## Discussion

As compared with normo-uricemic patients, hyperuricemic patients were more frequently male, with a more polyarticular and less axial PsA presentation, a more destructive radiographic involvement, more cardiovascular and renal comorbidities, and poorer response to PsA treatment. In addition, median SU level was higher with destructive than non-destructive PSA. Moreover, SU level was measured more frequently in patients with destructive than non-destructive PSA. These latter data may be explained by more frequent PsA flares with destructive than non-destructive PSA and more frequent measurement of SU level in this case to search for a differential diagnosis such as gout.

Our cohort of patients with PsA is mainly comparable to the known epidemiology of PsA [2, 21–23]. There were some notable differences: 46% of our patients had polyarticular PsA as compared with 20% in the literature [2]. This situation could be explained by an inclusion bias because all our patients were tested for uricemia in that a polyarticular presentation could suggest the diagnosis of gout. Only 14.5% of our patients had enthesitis (vs 30 to 50% in the literature), and 26% had dactylitis (vs 40 to 50% in the literature) [2]. This finding could be explained by the retrospective nature of our study, which may lead to lack of clinical data. There are no detailed data on the frequency of erosive PsA. The severity of PsA may have been overestimated in our cohort because of the inclusion criteria in our tertiary referral hospitals. Our rate of destructive involvement of PsA reached 42%, which is comparable to a recent Italian study (34.7% of 492 patients) [24].

The prevalence of gout in the French general population is 0.9% [25] and was 4.9% for men and 1.2% for women in a US cohort [10]. In our cohort of patients with PsA, the prevalence was 6.2% (only men), which is higher than in the general population. A few reports described patients with gout and psoriasis [26–29] and the coexistence of gout and PsA [27, 30–32]. These studies highlight the many common, sometimes confusing, risk factors shared by gout and PsA.

The prevalence of hyperuricemia in Europe reaches up to 25% [33]. In our PsA cohort, this prevalence was 30%. These data are similar to the 30.6% prevalence of PsA in a Chinese cohort [9] and the 31.9% prevalence in a prospective Canadian cohort [5]. In contrast, in a Japanese case–control study, the prevalence of hyperuricemia in PsA patients was only 22% [34]. However, SU level thresholds were not the same in all of these studies; hyperuricemia was defined as above 360 µmol/L for women and 420 µmol/L for men in the Chinese study, above 360 µmol/L for women and 450 µmol/L for men in the Canadian study, and above 330 µmol/L for women and 468 µmol/L for men in the Japanese study. In our study, statistical analyses were performed with different SU level thresholds, including 360 µmol/L for women and 420 µmol/L for men. The findings remained the same, but the small number of patients in our cohort with median SU level > 420 µmol/L would have implied lack of power in statistical analysis.

Little is known about the stability of the measurement in the same person over time, and some studies are limited because they measured SU level at a single time point [35]. However, SU level may vary depending on several factors [16, 36–38]. To overcome this eventual bias, we used the median of all SU measures for each patient. Indeed, we did not want to classify as “hyperuricemic” a patient who would have repeated normal SU levels but an unusual single high level, which could have been linked to confusing factors. As an example, kidney failure, both acute [39] and chronic [40], is known to be associated with hyperuricemia and gout, regardless of which is the cause or consequence. To avoid this “renal” bias, the presence or not of co-existing acute renal impairment was specified if only one uricemia sample was available and was ≥ 360 µmol/L. Moderate or severe chronic renal failure was included in both univariate and multivariable analyses. Another obvious example could be the history of hypo-uricemic treatment. In our cohort, 3 of 14 patients with a history of treatment with allopurinol belonged to the normo-uricemic group because their median SU level was above the threshold. One had a history of gout attack and may have been misclassified. The 2 others received treatment for only isolated hyperuricemia; we did not exclude them because of no accurate SU level. Finally, cutaneous psoriasis extent has been described to be a hyperuricemia risk factor [41]. In our analyses of psoriasis skin and nail involvement, only 2 patients had erythrodermic psoriasis. Most patients no longer had psoriasis with PsA treatment or had mild psoriasis. In that context, even if psoriasis area and severity index score had not been collected, it would have been useless.

Our study has some limitations. The retrospective data limit its power. Our inclusion criteria could have overestimated the severity of PsA because the use of ICD-10 coding allowed us to include only hospitalized patients, thus implying Berkson’s bias [42]. In the same way, the prevalence of hyperuricemia and gout may have been overestimated in our population because of the inclusion of patients with at least one SU sample. In addition, the absence of disease activity as a means of assessing remission in patients is a notable limitation. This is indeed due to the retrospective design of our study.

The main strength of our study is that it is the first to specifically evaluate PsA in terms of SU level. A large number of patients were included, for which we also had little missing data because of deep reading of medical files.

**Fig. 1:**
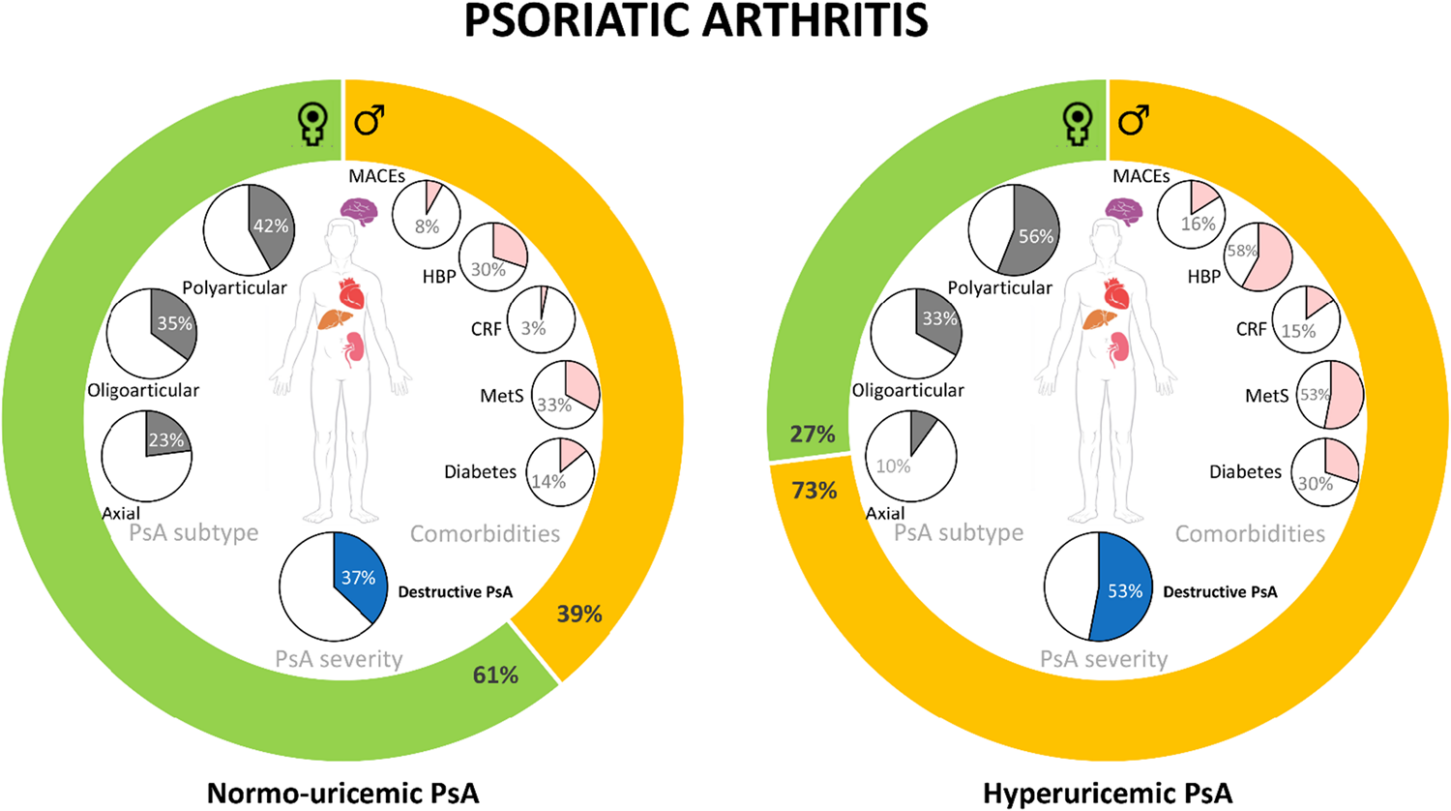
Description of normo- and hyperuricemic psoriatic arthritis. CRF, moderate to severe chronic renal failure; MACEs, major adverse cardiovascular events; HBP, high blood pressure; MetS, metabolic syndrome; PsA, psoriatic arthritis

The present study allows for distinguishing 2 distinct PsA profiles according to their association with hyperuricemia (Fig. 1).

Our results support a potential worsening impact of MSU crystals on PsA, previously named psout [1]. The existence of this entity would help in the medical care of patients. In practice, a flare of PsA must be determined as not a gout attack. Beyond that, knowledge of hyperuricemic PsA should lead to even more personalized medicine.

**Fig. 2:**
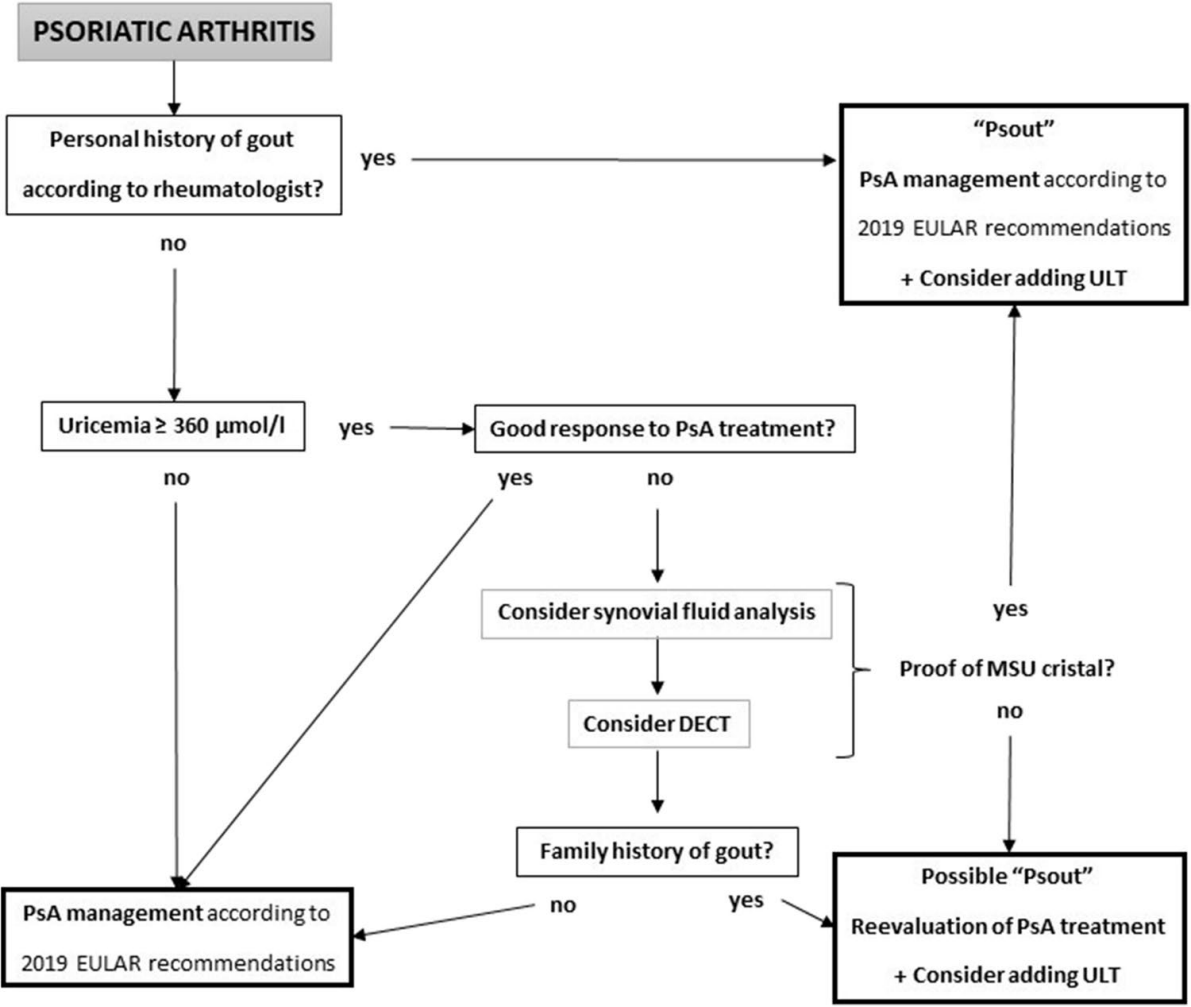
Recommendations for the diagnosis of psout and management of PsA with or without hyperuricemia. DECT, dual energy computed tomography; MSU, monosodium urate. Because of the retrospective method of our study, these recommendations should be taken with caution

Here, we propose recommendations for the diagnosis of psout and management of PsA with or without hyperuricemia (Fig. 2).

To confirm this viewpoint, new studies, especially prospective and interventional ones, could help better understand the role of hyperuricemia in PsA.

## Conclusion

Patients with hyperuricemic PsA show poorer response to PsA treatment and more peripheral and destructive joint damage than do normo-uricemic patients.

## Data Availability

All data generated or analyzed during this study are included in this published article.

## Declarations

### Patient involvement

Patients were not involved in the design, conduct, reporting, and dissemination plans of this article.
